# Effect of Fecal Microbiota Transplantation on Non-Alcoholic Fatty Liver Disease: A Randomized Clinical Trial

**DOI:** 10.3389/fcimb.2022.759306

**Published:** 2022-07-04

**Authors:** Lanfeng Xue, Zhiliang Deng, Wenhui Luo, Xingxiang He, Yu Chen

**Affiliations:** ^1^ Department of Gastroenterology, The Seventh Affiliated Hospital of Southern Medical University, Foshan, China; ^2^ Department of Gastroenterology, the First Affiliated Hospital of Guangdong Pharmaceutical University, Guangzhou, China

**Keywords:** fecal microbiota transplantation, non-alcoholic fatty liver disease, gut microbiota, lean NAFLD, obese NAFLD

## Abstract

**Background and Aims:**

The clinical efficacy of fecal microbiota transplantation (FMT) in patients with non-alcoholic fatty liver disease (NAFLD) and the variant effects of FMT on lean and obese NAFLD patients remain elusive. Our study aimed to determine the clinical efficacy and safety of FMT for patients with NAFLD, elucidating its different influences on lean and obese patients with NAFLD.

**Methods:**

We performed a randomized and controlled clinical trial. Patients in the non-FMT group were administered oral probiotics. In the FMT group, patients were randomized to receive FMT with donor stool (heterologous) *via* colonoscopy, followed by three enemas over 3 days. Both groups were also required to maintain a healthy diet and keep regular exercise for more than 40 min every day. They returned to the hospital for reexamination 1 month after treatment.

**Results:**

FMT can decrease the fat accumulation in the liver by improving the gut microbiota dysbiosis, thus attenuating fatty liver disease. Significant differences in the clinical features and gut microbiota between lean and obese NAFLD patients were unveiled. Moreover, FMT had better effects on gut microbiota reconstruction in lean NAFLD than in obese NAFLD patients.

**Conclusions:**

FMT could successfully improve the therapeutic effects on patients with NAFLD, and its clinical efficacy was higher in lean NAFLD than in obese NAFLD patients.

## Introduction

The morbidity of non-alcoholic fatty liver disease (NAFLD) has been increasing over the years, making it the most common chronic liver disorder across the globe, with an estimated worldwide prevalence of 25% (Chalasani et al., 2012). Risk factors including genetics, diet, obesity, and gut microbiota may cause a simple fatty liver to progress into non-alcoholic steatohepatitis (NASH), hepatic fibrosis, hepatic cirrhosis, and even hepatic carcinoma by promoting hepatocellular damage and inflammation (Adams and Angulo, 2005). It is estimated that NAFLD would become the most common indication for liver transplantation by 2030 (Danford and Lai, 2019). Currently, lifestyle changes remain the main treatment approach for patients with NAFLD (Pelusi and Valenti, 2019), including a healthy diet, exercise, and weight loss. However, for various reasons, a lot of patients find it difficult to persist with lifestyle-changing habits. NAFLD has become a serious threat to public health, and unfortunately, there is still no specific treatment for it.

Clarification of the pathogenesis and exploration of novel therapeutic approaches are of vital importance in the prevention of and interventions for NAFLD. In recent years, bourgeoning literature has shown that alterations in the gut microbiota, or the imbalance of intestinal bacteria, can contribute to microbiota dysbiosis, which plays an essential role in the pathogenesis of NAFLD (Hagymási et al., 2018; Lau and Wong, 2018; Sharpton et al., 2019). The underlying pathways involved in liver damage mainly include the activation of pathogen-associated molecular patterns (PAMPs) and damage-associated molecular patterns (DAMPs). Dysbiosis increases gut permeability to bacterial products and hepatic exposure to injurious substances *via* PAMPs or DAMPs, thus triggering hepatic inflammation and fibrosis (Leung et al., 2016). Multiple recent studies have proposed that an altered gut microbiota may be a new therapeutic approach for NAFLD (Safari and Gérard, 2019).

Fecal microbiota transplantation (FMT) is a novel approach to restoring and reconstructing the intestinal microecological balance and diversity. Apart from infection with *Clostridioides difficile* (Surawicz et al., 2013), FMT has demonstrated its efficacy in the treatment of several diseases, including metabolic diseases, tumors, autoimmune diseases, and hepatic encephalopathy (de Groot et al., 2017; Vaughn et al., 2019; Meighani et al., 2020). Several animal-based studies have shown that, by altering gut microbiota dysbiosis, FMT could effectively improve the manifestations of NAFLD (Zhou et al., 2017; Soderborg et al., 2018; García-Lezana et al., 2018). Therefore, the application of FMT to patients with NAFLD has become an attractive trend. Until now, only a few studies on the efficacy of FMT in the clinical treatment of NAFLD have been reported. A randomized control trial has shown that FMT has the potential to reduce small intestinal permeability in patients with NAFLD (Craven et al., 2020). Witjes et al. (2020) also indicated that FMT from healthy donors could affect hepatic gene expression and the plasma metabolites involved in inflammation and lipid metabolism, highlighting the crosstalk between gut microbiota composition and NAFLD.

NAFLD occurs not only in the obese population but also in lean people (2004). In the past, the characteristics, risk factors, and pathogenesis of NAFLD pointed to obese NAFLD patients; therefore, lean NAFLD patients were easily ignored, and this could lead to cryptogenic liver disease. Intriguingly, a recent study has demonstrated the differences in the epidemiological characteristics, clinical features, and pathological characteristics between lean and obese NAFLD patients (Lee et al., 2020). Moreover, compared with that in obese NAFLD patients, the treatment for lean NAFLD patients is relatively limited and challenging. Therefore, an in-depth study of the pathogenesis and treatment strategies for lean NAFLD patients is urgently required.

In the present study, we performed a clinical study mainly aimed at FMT for NAFLD, thus comparing the therapeutic efficacy between lean and obese NAFLD patients.

## Materials and Methods

### Subjects and Study Design

Patients who met the criteria of the American Association for the Study of Liver Disease (AASL) Guidelines for the Diagnosis and Treatment of Non-Alcoholic Fatty Liver Disease (2018 revision) were recruited in this study. According to the World Health Organization (WHO) Asia-Pacific Guidelines (Chalasani et al., 2018), NAFLD patients with body mass index (BMI) ≥25kg/m^2^ were referred to as obese NAFLD and those with BMI <25kg/m^2^ considered as lean NAFLD patients. The severity of fatty liver was based on the FibroScan fat attenuation degree: mild fatty liver (fat attenuation degree, <260 dB/m), moderate fatty liver (fat attenuation degree, 260–290 dB/m), and severe fatty liver (fat attenuation degree, >290 dB/m). The exclusion criteria were as follows: 1) severe complications or other critical diseases (for instance, heart, lung, and kidney failure, serious mental illness, etc.); 2) antibiotic intake within 1 month; 3) hazardous alcohol intake; and 4) medical history of drugs affecting liver function.

### Clinical and Laboratory Evaluations

**Figure 1:**
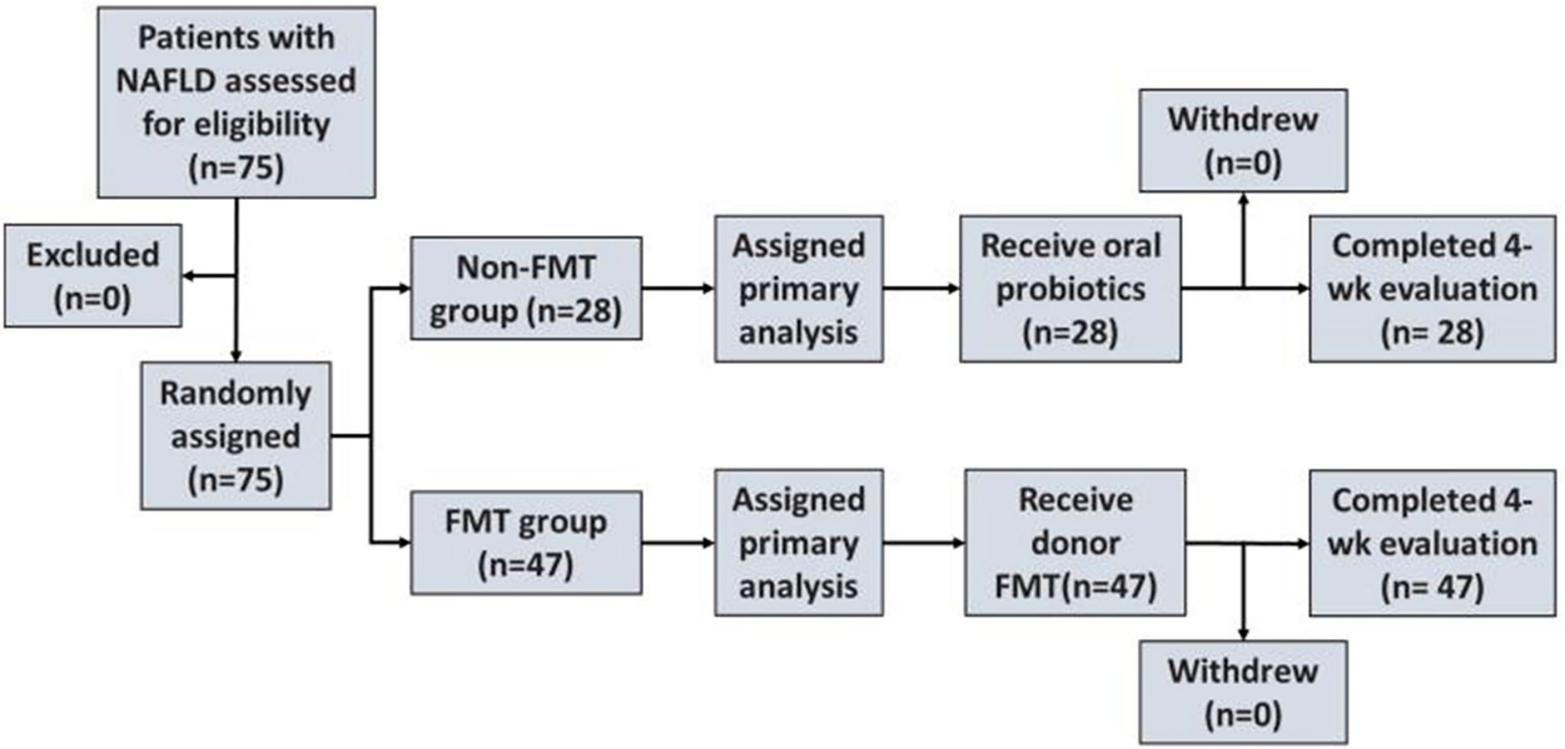
Enrollment and outcomes. NAFLD, non-alcoholic fatty liver disease; FMT, fecal microbiota transplantation.

The flowchart of the patients with NAFLD in the FMT trial is described in **Figure 1**.

#### Comparison of NAFLD Patients Treated With FMT and Non-FMT

Patients with NAFLD were randomly divided into an FMT group and a non-FMT group (traditional treatment group) based on the wishes of the participant (**Figure 1**). Feces for the FMT group were obtained from healthy undergraduate donors (Guangdong Pharmaceutical University) and evaluated using comprehensive physical examinations. The preparation and the transplantation of fecal microbiota were performed as previously described. Briefly, 100 g of feces with 500 ml of 0.9% saline was used to prepare the homogeneous fecal suspension from healthy donors. The fecal suspension was microfiltered using an automatic machine. Afterward, the filtered fecal suspension was centrifuged at 1,100 × *g* for 3 min at room temperature. Subsequently, 100 ml of saline was added to the microbiota pellet obtained from 100 g of feces.

In the non-FMT group, the patients were administered oral probiotics (a *Bifidobacterium* viable preparation and *Lactobacillus acidophilus* capsules). In the FMT group, each patient received a total of 200 ml of fresh bacteria solution per day, for 3 days in total (He et al., 2017). Both groups were also required to maintain a healthy diet (plenty of vegetables, low fat, low sugar, and low salt) and keep regular exercise for more than 40 min every day. They returned to the hospital for reexamination 1 month after treatment.

#### Comparison of Lean NAFLD Patients and Obese NAFLD Patients in the FMT Group

Patients in the FMT group were divided into obese NAFLD and lean NAFLD groups according to their BMIs. The two groups of related indicators were then compared.

### Clinical Data Collection

#### General Information

Patients’ general information (including gender, age, height, and weight), medical histories, and examination results [such as fasting blood sugar, aspartate aminotransferase (AST), blood lipid, fasting insulin, lipopolysaccharide, and FibroScan] were collected. Improvements in the blood lipid, blood glucose, blood pressure, fatty liver severity, and other indicators are important indicators for the evaluation of the therapeutic effect, especially the fatty deposition in the liver. The efficacy of FMT in the treatment of patients with NAFLD was verified by comparing the indexes of the FMT group before and after treatment and by analyzing the differences between the FMT and non-FMT groups.

#### Fecal Collection, DNA Extraction, MiSeq Sequencing, and 16S rRNA Gene Sequencing

In the FMT group, 10 samples of fresh feces were randomly collected before and after treatment. Ten samples from healthy donors were collected and taken as the control group. The time points of the fecal collection were set as the treatment day and 1 month after FMT. All samples were stored in the refrigerator at −80°C after collection until DNA extraction. Microbial DNA was extracted using the E.Z.N.A.^®^ Soil DNA Kit (Omega Biotek, Norcross, GA, USA) according to the manufacturer’s instructions. The quality and the concentration of DNA were checked using a spectrophotometer (NanoDrop™ 2000; Thermo Fisher Scientific, Wilmington, DE, USA). Bacterial 16S ribosomal RNA (rRNA) gene fragments (V3–V4) were amplified from the extracted DNA using primers 338F (5′-ACTCCTACGGGAGGCAGCAG-3′) and 806R (5′-GGACTACHVGGGTWTCTAAT-3′) and the following polymerase chain reaction (PCR) conditions: 30 s at 95°C, 30 s at 55°C, and 45 s at 72°C for 25 cycles. PCRs were carried out with 4 ml of 5× TransStart FastPfu buffer, 2 ml of 2.5 mM deoxynucleoside triphosphates, 0.8 ml of each primer (5 mM), 0.4 ml TransStart FastPfu DNA polymerase, 10 ng of extracted DNA, and, finally, with double-distilled water to take the volume up to 20 ml. Agarose gel electrophoresis was undertaken to ascertain the size of the amplicons. The latter were subjected to paired-end sequencing on the MiSeq sequencing platform (Illumina, San Diego, CA, USA) using PE300 chemical from Majorbio BioPharm Technology (Shanghai, China).

**Figure 2:**
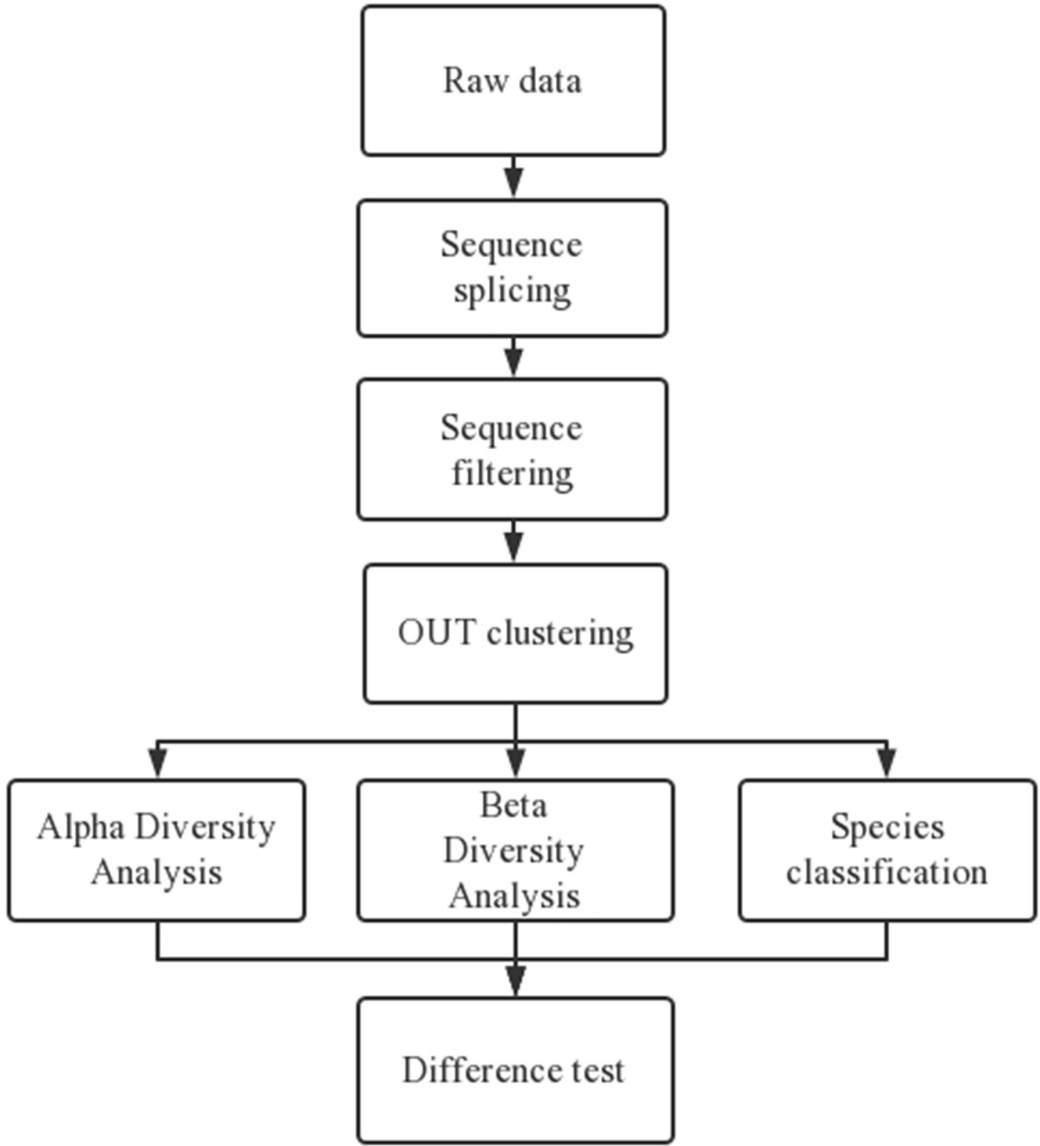
Specific 16S rRNA analysis steps.

Subsequently, the microbiota underwent 16S rRNA gene sequencing to observe the characteristics and changes in the gut microbiota (Klindworth et al., 2013; Zhong et al., 2021). The specific 16S rRNA analysis steps are represented in **Figure 2**. The original image data produced by high-throughput sequencing were transformed into the sequencing data according to the base calling analysis, and the results were stored in a fastq file format. Thereafter, the original data were spliced and filtered. Finally, comparative analyses of the alpha diversity, beta diversity, and species classification were applied. Analyses of the 16S rRNA microbiome sequencing data were processed using the online platform Majorbio Cloud (www.majorbio.com/), a one-stop, comprehensive bioinformatic platform for multiomics analyses (Ren et al., 2022). The platform consists of three modules: preconfigured bioinformatic pipelines, cloud toolsets, and online omics courses.

High-quality sequences were clustered into operational taxonomic units (OTUs) at 97% sequence identity. After obtaining representative OTU sequences, high-quality sequencing data on the length distribution of all samples were determined. A certain number of sequences were randomly selected from each sample to reach a uniform depth for the prediction of observed OTUs and their relative abundances (Heck Jr., et al. 1975; Kemp and Aller, 2004). To calculate the alpha diversity, we rarified the OTU table and calculated the Chao1 index to estimate species richness in order to describe community diversity (Chao, 1984). Additionally, principal coordinates analysis (PCoA) was employed for the analysis of the beta diversity. The more similar the community composition of the samples, the closer their distance in the PCoA diagram. Species and community composition analyses were represented by Venn diagrams and bar plots. Venn diagrams were used to count the number of species according to the uniqueness of each group and the OTUs shared between the groups.

### Statistical Method

Continuous variables were presented as the mean ± standard deviation, and comparisons between groups were performed with paired Student’s *t*-test or the Mann–Whitney test. Categorical variables and enumeration data were presented as counts and proportions, and the groups were compared using chi-square tests. The Kruskal–Wallis test was used to compare the changes in the gut microbiota between groups. The 16S rRNA microbiome sequencing data were analyzed using the free online platform Majorbio Cloud. Firstly, we accessed the bioinformatics pipelines, selected the microbial diversity QIIME2 process, and input the original data. Afterward, the high-quality sequences were denoised using the DADA2 plug-in (Callahan et al., 2016) in the QIIME2 (version 2020.2) (Bolyen et al., 2019) pipeline using recommended parameters. The DADA2-denoised sequences were named “amplicon sequence variants” (ASVs). Lastly, the community diversity, community composition, and species differences were analyzed. A *p-*value <0.05 indicated statistical difference.

## Results

Eighty patients with NAFLD were included; among them, five patients withdrew from the clinical trial due to personal reasons. A total of 75 cases were finally recruited and were divided into the FMT group (*n* = 47) and the non-FMT group (*n* = 28). In addition, 10 healthy individuals were included as the control group. In the FMT group, 15 cases (31.9%) were lean and 32 cases (68.1%) were obese.

We compared the baseline information including gender, age, BMI, blood glucose, insulin, etc. No statistical differences were found between the FMT group and the non-FMT group (**Supplementary Table S1**).

The results of the blood lipid and liver function tests before and after treatment in the FMT group showed no statistical difference (*p* > 0.05) (**Supplementary Table S2**). Expectedly, the hepatic fat attenuation evaluated by FibroScan was significantly reduced after FMT (*p* < 0.05).

Between the FMT and non-FMT groups, there were no statistical differences in the blood lipid, liver function, and fat attenuation results before treatment (*p* > 0.05). After FMT, no statistical differences were found in the blood lipid and liver function results between the FMT and non-FMT groups. The mean fat attenuation degrees decreased from 278.3 to 263.9 dB/m in the FMT group (*p* = 0.049), while the liver fat attenuation values increased from 265.5 to 282.5 dB/m in the non-FMT group (*p* < 0.01).

We next checked the characteristics and the changes in the gut microbiota (**Table 1**). Compared to healthy individuals, patients with NAFLD before the FMT had lower Chaol indexes [prior to FMT (pri-FMT) vs. healthy group, *p* < 0.05], suggesting a lower abundance of the gut microbiota in patients with NAFLD. On the other hand, there was no statistical difference in the Chaol indexes between NALFD patients post-FMT (po-FMT) and healthy individuals (*p* > 0.05), indicating that the impaired abundance of the gut microbiota had been improved after FMT.

**Table 1:** Comparison of the Chao1 indexes between healthy donors and patients with non-alcoholic fatty liver disease (NAFLD).

	Pri-FMT (*n* = 10)	Po-FMT (*n* = 10)	Healthy group (*n* = 10)	*p*-value (pri-FMT vs. healthy group)	*p*-value (po-FMT vs. healthy group)
Chao1	72.4	85	102.3	0.04	0.438

A total of 30 samples were analyzed, with an average number of sequences of 12,675.

pri-FMT, prior to fecal microbiota transplantation; po-FMT, post-fecal microbiota transplantation.

**Figure 3:**
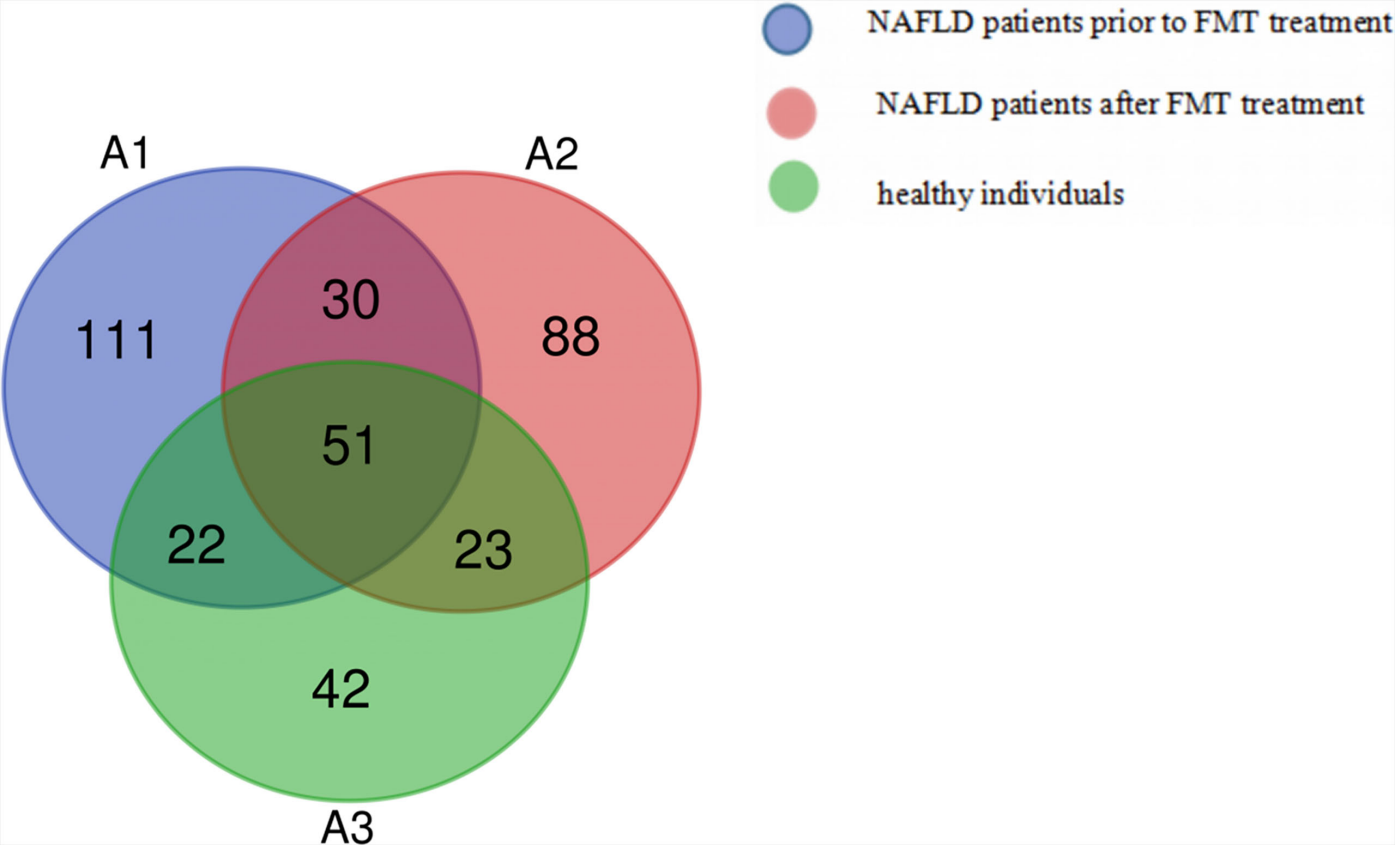
Venn diagram of patients with non-alcoholic fatty liver disease (NAFLD) and healthy individuals.

**Figure 4:**
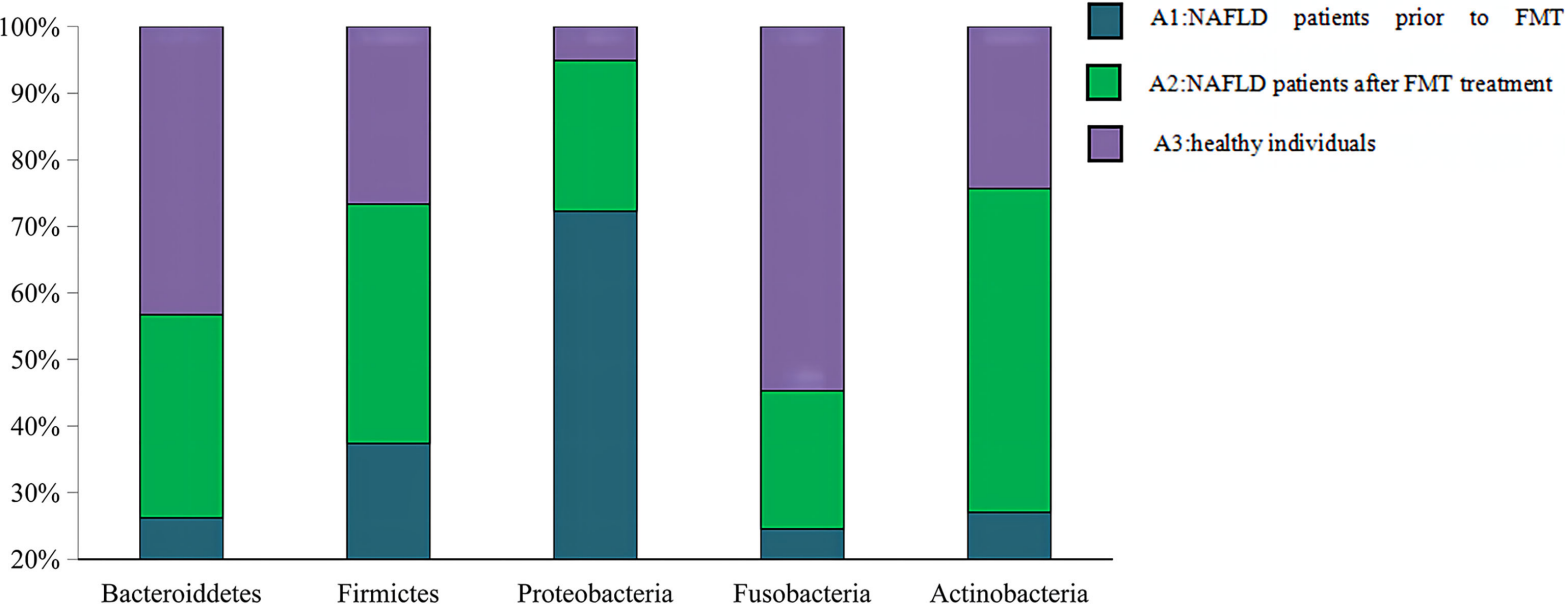
Percentage of each phylum in patients with non-alcoholic fatty liver disease (NAFLD) and healthy individuals. An increasing trend of Bacteroidetes and Bacteroidetes/Firmicutes (B/F) ratio was observed from the pre-fecal microbiota transplantation (pri-FMT) group to the healthy group.

The Venn diagram based on the number of OTUs is shown in **Figure 3**. At the phylum level, the distribution of the gut microbiota in healthy individuals and in patients with NAFLD before and after FMT was observed. As shown in **Figure 4** and **Supplementary Table S3**, the proportions of Bacteroidetes were 35.1% before FMT, 40.9% after treatment, and 58.0% in the healthy group (pri-FMT vs. healthy group, *p* < 0.05; po-FMT vs. healthy group, *p* > 0.05). The Bacteroidetes-to-Firmicutes (B/F) ratios were 0.7 before FMT, 0.93 after treatment, and 1.54 in the healthy group (pri-FMT vs. healthy group, *p* < 0.05; po-FMT vs. healthy group, *p* > 0.05). Furthermore, patients with NAFLD before FMT had a significantly higher proportion of Proteobacteria than did healthy individuals (pri-FMT vs. healthy group, *p* < 0.05).

The family-level data are shown in **Supplementary Table S4**. Both Bacteroidetes and Muribaculaceae belong to the Bacteroidetes family, of which the pri-FMT group had a significantly lower proportion than the po-FMT or the healthy group (pri-FMT vs. healthy group, *p* < 0.05; pri-FMT vs. po-FMT, *p* < 0.05). There was no significant difference in certain bacterial contents between the po-FMT and healthy groups (po-FMT vs. healthy group, *p* > 0.05). The Christensenellaceae, a member of the Firmicutes family, was found to be significantly lower in the pri-FMT group compared to the po-FMT and healthy groups (pri-FMT vs. healthy group, *p* < 0.05; pri-FMT vs. po-FMT, *p* < 0.05). There was also no significant difference in this bacterial content between the po-FMT group and the healthy group (po-FMT vs. healthy group, *p* > 0.05). As another member of the Firmicutes family, the Ruminococcaceae also showed a rising trend in bacterial content from the pri-FMT to the healthy group. Compared to those in the healthy group, the bacterial contents in both pri-FMT and po-FMT groups were significantly lower (pri-FMT vs. healthy group, *p* < 0.05; po-FMT vs. healthy group, *p* < 0.05). However, no statistical difference between the pri-FMT and po-FMT groups was observed (pri-FMT vs. po-FMT, *p* > 0.05).

The genus-level data are shown in **Supplementary Table S5**. Compared to the healthy group, the pri-FMT group had lower proportions of the following 14 types of bacteria: *Bacteroides*, *Christensenellaceae R-7*, *Metagenome*, *Ruminococcus 1*, *Tyzzerella 3*, [*Eubacterium*] *coprostanoligenes group*, [*Eubacterium*] *ruminantium group*, *Intestinimonas*, *Mitsuokella*, *Rikenellaceae RC9 gut group*, *Roseburia*, and *Subdoligranulum* (pri-FMT vs. healthy group, *p* < 0.05). On the other hand, *Escherichia–Shigella* were more abundant in the pri-FMT group, but less in the po-FMT group (pri-FMT vs. healthy group, *p* < 0.05; pri-FMT vs. po-FMT, *p* < 0.05).

We set the lean NAFLD patients before and after FMT as B1 and C1 groups and the obese NAFLD patients before and after FMT as B2 and C2 groups, respectively. As shown in **Supplementary Table S6**, there was a significant difference in the prevalence of hyperlipidemia between the two groups (B1 vs. B2, *p* = 0.037). The BMIs of obese NAFLD patients were significantly higher than those of lean NAFLD patients (B1 vs. B2, *p* < 0.001), while there was no significant difference in BMI after FMT treatment in both groups (B1 vs. B2, *p* > 0.05).

As shown in **Supplementary Table S6**, both the average fasting insulin and insulin resistance index of the NAFLD patients in the obesity group were significantly higher than those in the lean group (B1 vs. B2, *p* < 0.05). However, the average triglyceride (TG) level of the NAFLD patients in the lean group was higher than that of patients in the obesity group (B1 vs. B2, *p* = 0.038).

**Figure 5:**
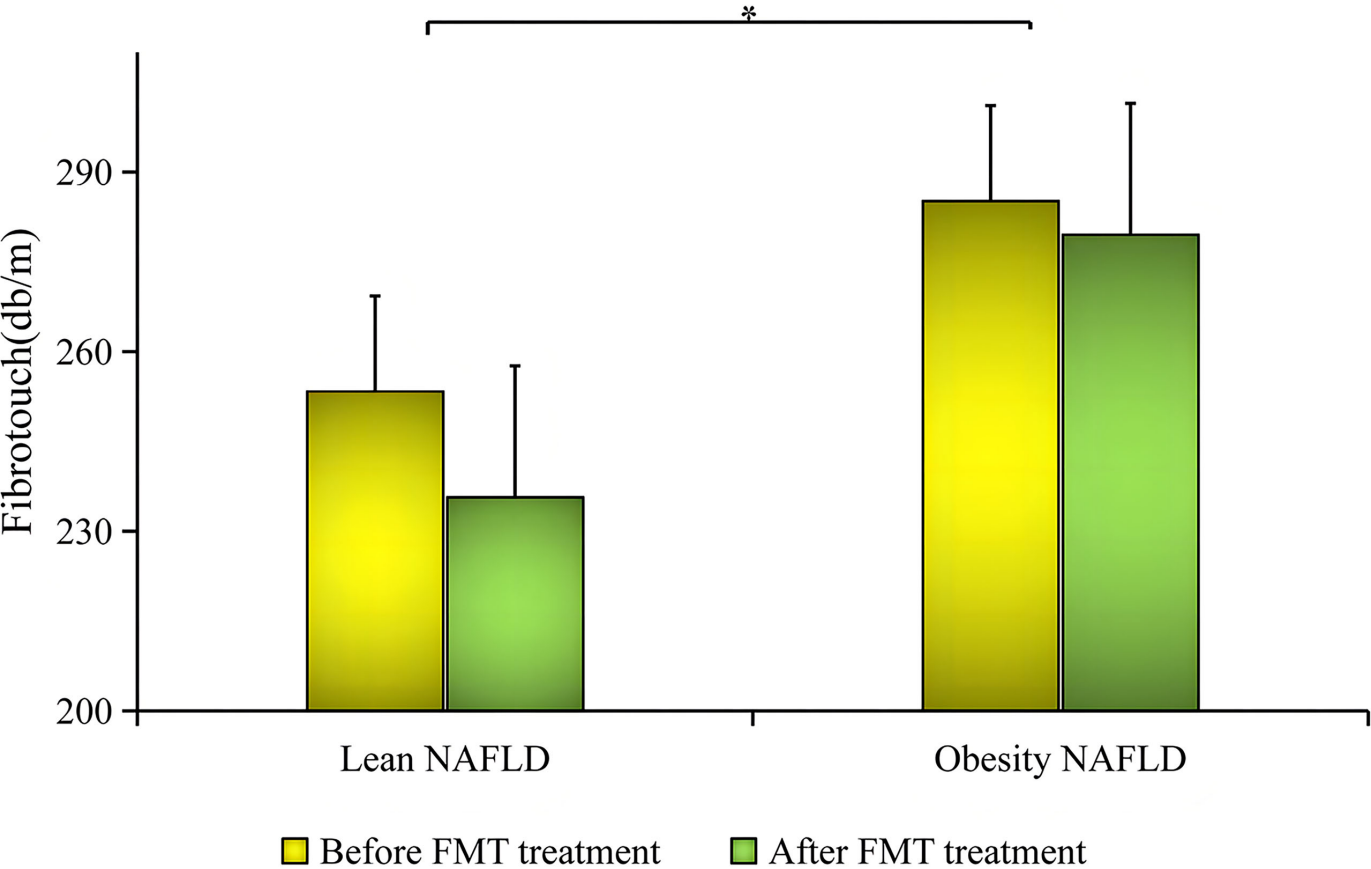
Comparison of the decrease in fat attenuation before and after fecal microbiota transplantation (FMT) between the two groups. * meaning Lean NAFLD vs obese NAFLD had statistical difference, P < 0.05.

**Figure 6:**
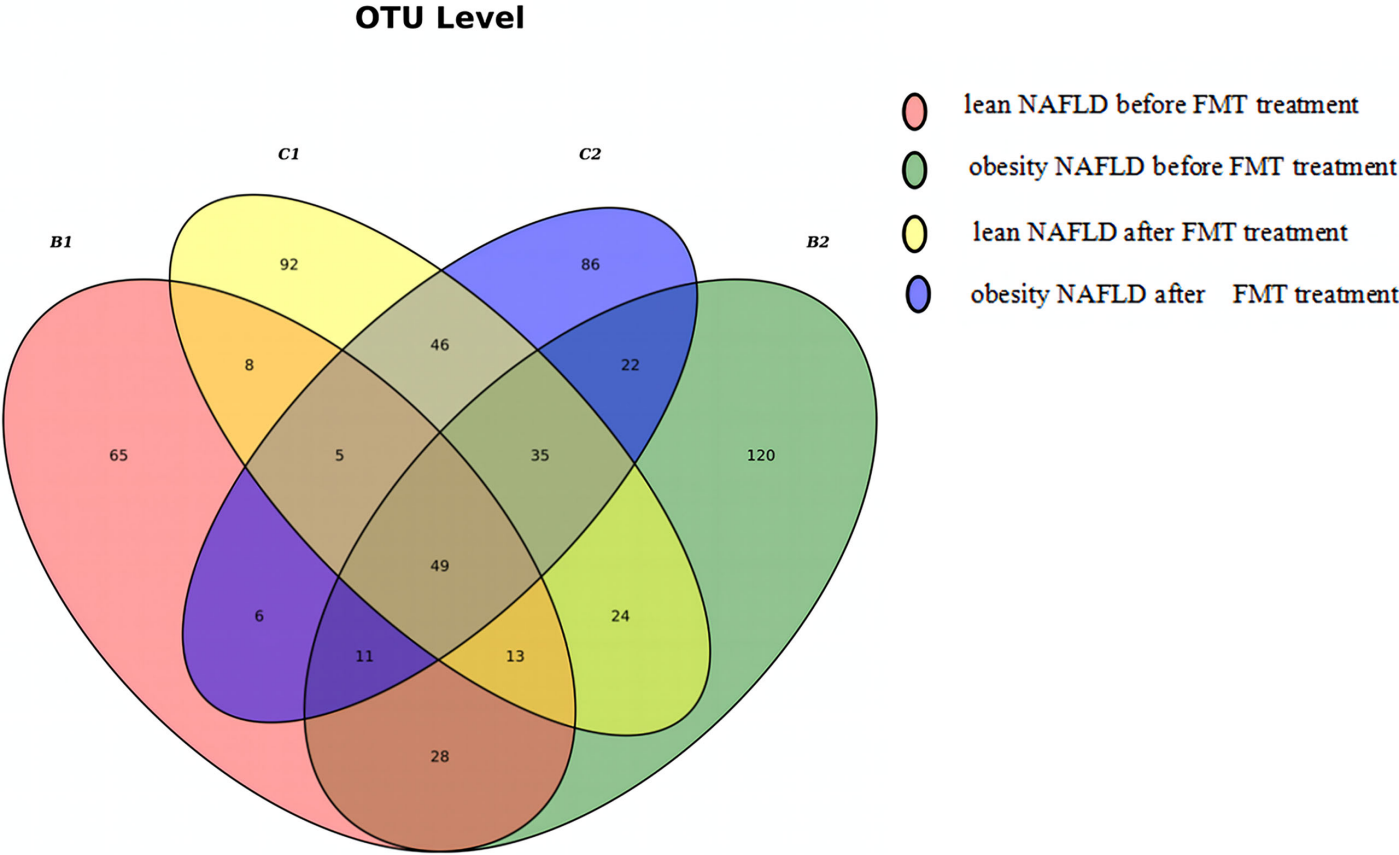
Venn diagram of lean and obese patients with non-alcoholic fatty liver disease (NAFLD) before and after fecal microbiota transplantation (FMT). A total of 654 operational taxonomic units (OTUs) were detected in the four groups, of which 101 OTUs were shared by B1 and B2 and 135 OTUs were shared by C1 and C2. One hundred ninety-seven different OTUs were found between C1 and B1, while 175 OTUs were detected in C2 and B2.

As shown in **Supplementary Table S6** and **Figure 5**, before FMT, the average fat attenuation of the NAFLD patients in the lean group was 253.4 ± 36.2 dB/m, which was significantly lower than that of patients in the obesity group (285.2 ± 42.3 dB/m), indicating that it was more serious in the lean group than in the obesity group (B1 vs. B2, *p* = 0.025). On the other hand, after FMT, the average fat attenuation of lean NAFLD patients decreased to normal levels (235.7 ± 24.5 dB/m), while that of obese NAFLD patients barely changed (279.5 ± 44.5 dB/m). Importantly, the statistical difference was significant, as shown in **Figure 6** (lean NAFLD vs. obese NAFLD patients, *p* = 0.029).

As shown in **Figure 6**, the Venn diagram was drawn according to the number of OTUs detected in the four groups. Taken together, it suggested that FMT had a greater impact on the microbial community structure in lean NAFLD than in obese NAFLD patients. The 16S rRNA gene sequencing data of patients with NAFLD were regrouped according to the BMI before FMT: 10 cases of NAFLD, including four cases in the lean group and six cases in the obesity group. A total of 20 samples were tested and an average of 12,669 sequences read for each sample. The results showed that the mean numbers of OTUs were 63.5 (B1), 78.3 (B2), 96.5 (C1), and 75.8 (C2), indicating that the OTU numbers in lean NAFLD patients increased after FMT, while those in obese NAFLD patients slightly decreased after FMT.

**Figure 7:**
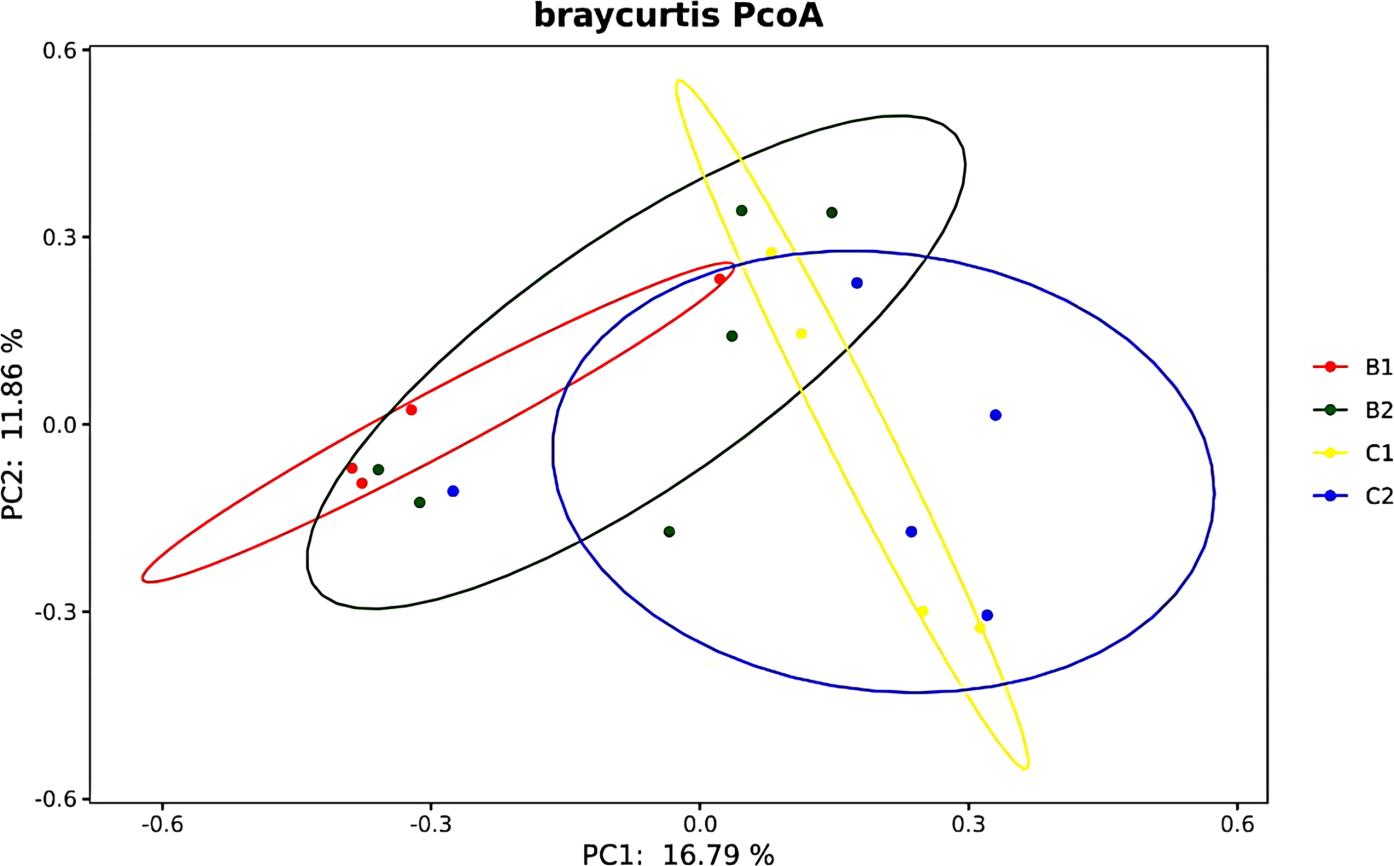
Diagram of the Bray–Curtis distance principal coordinate analysis.

The PCoA map was drawn according to the Brady–Curtis distance between the samples. As shown in **Figure 7**, the gut microbiota community structure of patients with NAFLD (B1 + B2) was changed after FMT (C1 + C2). Analysis of similarities (ANOSIM) showed that there was no significant difference in the gut microbiota community structure between the lean NAFLD and obese NAFLD patients before FMT (B1 vs. B2, *p* = 0.604, *r* = −0.04). However, there was a moderate difference in the gut microbiota community structure in lean NAFLD patients before and after FMT (B1 vs. C1, *p* = 0.018, *r* = 0.590), while it was not significant in obese NAFLD patients (B2 vs. C2, *p* = 0.256, *r* = 0.059). The results of permutational multivariate analysis of variance (PERMANOVA) were in line with those of ANOSIM (B1 vs. B2, *p* = 0.616; B1 vs. C1, *p* = 0.028; B2 vs. C2, *p* = 0.269), suggesting that NAFLD patients in the lean group were more sensitive to FMT than those in the obese group.

The relevant results were analyzed and are shown in **Supplementary Table S7** (B1 vs. B2). At the phylum level, the abundance of Actinobacteria in lean NAFLD patients was significantly higher than that in obese NAFLD patients (*p* < 0.05). However, no significant difference in the gut microbiota was detected in both groups at the class level (*p* > 0.05). At the order level, the abundance of Desulfovibrionales in lean NAFLD patients was significantly elevated than that in obese NAFLD patients (*p* < 0.05). At the family level, the abundance of Tannerellaceae in lean NAFLD was increased compared to that in obese NAFLD patients (*p* < 0.05). At the genus level, the abundance rates of *Prevotella 2*, *Lachnospiraceae nk4a136 group*, *Lachnospiraceae nd3007 group*, [*Eubacterium*] *Coprostanoligenes*, *Mitsuokella*, and *Fusicatenibacter* in lean NAFLD patients were significantly lower than those in obese NAFLD patients (*p* < 0.05). At the species level, the abundances of *Bacteroides coprocola DSM 17136* and uncultured *Roseburia* sp. in lean NAFLD were significantly higher than those in obese NAFLD patients (*p* < 0.001), while *Bifidobacterium longum* subsp. *longum* was significantly higher in lean NAFLD than in obese NAFLD patients (*p* < 0.001).

The relevant results are listed in **Supplementary Table S8** (B1 vs. C1 and B2 vs. C2). The following were significantly decreased in the gut microbiota of lean NAFLD patients after FMT: Actinobacteria, Desulfovibrionales, Bacteroidaceae, *Bacteroides*, and [*Ruminococcus*] *gnavus group* (B1 vs. C1, *p* < 0.05). On the contrary, the following were significantly upregulated in the gut microbiota of lean NAFLD patients after FMT: Selenomonadales, Veillonellaceae, *Prevotella 2*, [*Eubacterium*] *coprostanoligenes group*, [*Eubacterium*] *ruminantium group*, *human gut metagenome*, and *uncultured Roseburia* sp. (B1 vs. B2, *p* < 0.05). It was worth noting that the proportions of *Prevotella 2*, [*Eubacterium*] *coprostanoligenes group*, and [*Eubacterium*] *ruminatum group* before treatment were all 0%, which then significantly increased after FMT, while the proportion of [*Ruminococcus*] *gnavus group* before FMT was 2.9%, which then decreased to 0% after FMT. In addition, the proportion of Bacteroidaceae decreased from 40.6% to 35.7%, while that of *Bacteroides* decreased from 51.5% to 38.1% after treatment. Compared with healthy individuals (**Supplementary Table S5**), the proportions of Bacteroidaceae and *Bacteroides* in lean NAFLD patients before FMT were higher, but decreased after FMT. These results identified that gut dysbacteriosis can be induced due to the imbalanced ratio of Bacteroidaceae to *Bacteroides*.

In obese NAFLD patients, *Rikenella RC9 gut group* and *Alistipes* were significantly decreased after FMT (B2 vs. C2, *p* < 0.05), while *Ruminococcus 2* and *Prevotella 2* were significantly upregulated (B2 vs. C2, *p* < 0.05). Moreover, *Lactobacillus* was slightly increased after FMT (B2 vs. C2, *p* = 0.054).

## Discussion

Due to various factors, only a few patients could persist on keeping a healthy lifestyle to improve NAFLD. Sometimes, NAFLD worsens, which was fully shown in the non-FMT group. This phenomenon reflects the dilemma of the clinical treatment of NAFLD at the present stage. Clinically, patients with NALFD often present with gastrointestinal symptoms at the same time, verifying the interaction of the gut and liver. As shown in our study, FMT successfully improved fatty liver disease and had a better effect than the original treatment without FMT, confirming that the gut microbiota plays a key role in the pathogenesis of NAFLD. In addition, the manifestations of some of the NAFLD patients in the FMT group accompanied by chronic diarrhea and constipation in were alleviated after receiving FMT.

The gut microbiota and its metabolites have been proven to play a crucial role in the pathogenesis of NAFLD (Eckburg et al., 2005; Tilg, 2010). In this study, we confirmed that the proportion of Proteobacteria in patients with NAFLD before FMT was significantly higher than that in healthy individuals through 16S rRNA sequencing. Byndloss et al. (Byndloss and Bäumler, 2018) demonstrated that the expansion of Proteobacteria could be viewed as a microbial signature of epithelial dysfunction and dysbiosis. Similarly, several previous studies (Turnbaugh et al., 2006; John and Mullin, 2016; Johnson et al., 2017) have shown that a decrease in Bacteroidetes, an increase in Firmicutes, and an imbalance in B/F are all associated with obesity, especially the B/F imbalance. The imbalance of B/F can lead to excessive calorie intake and abnormal lipid accumulation. In terms of phylum, the B/F ratio of the gut microbiota in NAFLD was significantly disturbed. Thus, the imbalance of B/F may contribute to the onset of NAFLD.

Compared with healthy individuals, gut microbiota dysbiosis in patients with NAFLD can be characterized by reducing bacterial diversity and decreasing beneficial bacteria, which may cause metabolic disorders and opportunistic pathogen infection. The abundances of Bacteroidetes. rumen bacteria, Christensenellaceae, and other bacteria were significantly reduced in patients with NAFLD. Sophie et al. (Poeker et al., 2018) found that the gut microbiota dominated by Bacteroidetes and *rumen bacteria* produced more butyric acid, indicating that these two families are butyrate-producing bacteria. Butyric acid can protect the intestinal mucosal barrier (Wang et al., 2012) and enhance the body’s resistance to metabolic disorders caused by a high-fat diet (Ye et al., 2018). Christensenellaceae is widely found in human and animal intestines and mucous membranes. In terms of genus, 14 genera of bacteria were significantly reduced in the intestinal tract of NAFLD patients, most of which are probiotics related to maintaining normal metabolism, immunity, and intact mucosal barrier. Moreover, *Escherichia–Shigella* was significantly increased. Previous studies reported that *Escherichia–Shigella* is an opportunistic pathogen closely related to systemic inflammatory response and impaired intestinal barrier (Gao et al., 2018). Hence, decreased gut microbiota abundance, butyrate-producing bacteria, and increased inflammation-inducing bacteria are common mechanisms involved in NAFLD.

Obese NAFLD patients have characteristics of obesity and metabolic disorders, which causes the prevalence of hypertension higher than that in lean NAFLD patients. However, the levels of TG in lean NAFLD patients were significantly higher than those in obese NAFLD patients. We speculated that the main causes of abnormal TG levels in the two types of NAFLD may differ. The possible contributors may be gut microbiota disorder and hormone abnormality, causing abnormal lipid metabolism in lean NAFLD patients. The gut microbiota and its metabolites can act on different receptors and pathways to affect metabolism and cause or improve metabolic disorders (Chu et al., 2019). In this study, the effect of FMT on the treatment of lean NAFLD patients was more obvious than that in obese NAFLD patients. Therefore, we verified that the pathogenesis of lean and obese NAFLD patients was different, which may be the key reason. Owing to the good therapeutic effect of FMT on lean NAFLD patients, we hypothesized that gut microbiota disorder is more important in the pathogenesis of lean NAFLD than of obese NAFLD patients.

Differences in the gut microbiota may be one of the main reasons for the differences between lean and obese NAFLD patients. The number of “harmful bacteria” involved in the pathogenesis of fatty liver and related to inflammatory reactions was increased more obviously in lean than in obese NAFLD patients, and the number of bacteria negatively correlated with body weight and blood glucose was also increased (Delzenne and Bindels, 2018; Shikany et al., 2019). There were fewer beneficial bacteria in lean NAFLD patients to improve glucose and lipid metabolism and intestinal inflammation than in obese NAFLD patients (Li et al., 1998; Hamilton et al., 2018; Wang et al., 2019). In addition, *Proteus* was more abundant in obese NAFLD than in lean NAFLD patients. Proteobacteria is an intestinal symbiotic bacterium that can produce virulence factors related to gastrointestinal diseases and is closely related to gastrointestinal diseases, especially Crohn’s disease. It is suggested that the damage of the intestinal mucosal barrier in obese NAFLD patients can exacerbate the release of “harmful bacteria.”

In terms of clinical effects, FMT could improve NAFLD in both lean and obese patients, but the treatment effect of FMT on lean NAFLD patients was better than that on obese NAFLD patients. The characteristics of the gut microbiota between lean and obese patients with NAFLD varied in a wide range, and the patients in the two groups showed different responses to FMT. As is known, NAFLD in obese patients can be significantly improved after interventions of diet control, weight loss, and exercise. Therefore, we speculated that NAFLD in obese patients is mainly due to abnormal lipid deposition caused by excessive intake and obesity and leads to further damage of the gut microbiota. Improper lifestyle is the root cause of gut microbiota disorders in obese NAFLD patients. If the effect of lifestyle interventions such as diet and exercise on obese NAFLD patients is better than that of FMT, this hypothesis might be further verified. Lean NAFLD patients are characterized by normal BMI and blood lipid, with decreased number and diversity of beneficial gut microbiota, which was more sensitive to the therapeutic effects of FMT. We proposed that the main reason for the gut microbiota damage in lean NAFLD patients is the original lack of gut microbiota. FMT is equivalent to directly supplementing the source of gut microbiota. Therefore, the therapeutic effect of FMT on lean NAFLD patients was better than that on obese NAFLD patients.

## Conclusion

FMT can improve NAFLD by balancing gut microbiota disorder. FMT had better effects on the improvement of lean than of obese NAFLD patients. Lean and obese patients with NAFLD showed significant differences both in clinical manifestations and in the gut microbiota characteristics, mainly due to the different mechanisms of the gut microbiota disorder.

## Data Availability Statement

The datasets presented in this study can be found in online repositories. The name of the repository and accession number can be found below: NCBI; PRJNA782181.

## Ethics Statement

The studies involving human participants were reviewed and approved by the First Affiliated Hospital of Guangdong Pharmaceutical University. The patients/participants provided written informed consent to participate in this study.

## Author Contributions

LX, XH, and YC contributed to the study conception and design. LX, ZD, WL, and YC contributed to data acquisition, data analysis and interpretation, and writing of the article. LX, XHX, and YC contributed to the editing, reviewing, and final approval of the article. All authors contributed to the article and approved the submitted version.

## Funding

This study was support by The Dean’s Fund of the Seventh Affiliated Hospital of Southern Medical University (2021YZJJ005) and Medical scientific research project of Foshan Health Bureau (20220809A010452).

## Conflict of Interest

The authors declare that the research was conducted in the absence of any commercial or financial relationships that could be construed as a potential conflict of interest.

## Publisher’s Note

All claims expressed in this article are solely those of the authors and do not necessarily represent those of their affiliated organizations, or those of the publisher, the editors and the reviewers. Any product that may be evaluated in this article, or claim that may be made by its manufacturer, is not guaranteed or endorsed by the publisher.
