# Supplementary material for: Effect of Fecal Microbiota Transplantation on Non-Alcoholic Fatty Liver Disease: A Randomized Clinical Trial
Source: Front Cell Infect Microbiol. 2022 Jul 4;12:759306. doi: 10.3389/fcimb.2022.759306 (PMC9289257; doi:10.3389/fcimb.2022.759306)
Supplement: Supplementary file 1 [file Table_1.docx]

**Supplementary results section**

Supplementary Table 1. Baseline comparison between FMT group and non-FMT group

| characteristic | FMT group(n=47) | | Non-FMT group(n=28) | | *P value* |
| --- | --- | --- | --- | --- | --- |
|  | Before treatment | After  treatment | Before treatment | After  treatment |  |
| Gender (male) | 25（53.2%） | | 14（50%） | | ＞0.05 |
| Age | 57.3±13.4 | | 60.2±8.5 | | ＞0.05 |
| Hypertension | 23（48.9%） | | 12（42.8%） | | ＞0.05 |
| Type 2 diabetes | 15（31.9%） | | 8（28.5%） | | ＞0.05 |
| BMI (kg/m^2^) | 27.7±4.5 | 27.4±4.4 | 28.0±5.2 | 28.4±5.2 | ＞0.05 |
| PLT(10×10^9) | 231.8±66.7 | 229.8±60.1 | 221.4±73.7 | 223.9±75.6 | ＞0.05 |
| Hb (g/L) | 137.9±13.6 | 139.5±14.4 | 134.6±16.5 | 137.6±12.8 | ＞0.05 |
| Fasting blood glucose (mmol/L) | 5.3±1.2 | 5.3±1.3 | 5.6±1.6 | 5.8±1.9 | ＞0.05 |
| Fasting insulin （mU） | 15.1±8.9 | 13.4±6.7 | 12.6±6.0 | 15.8±8.9 | ＞0.05 |
| HOMA IR | 3.8±2.9 | 3.4±2.2 | 3.2±1.7 | 4.5±3.3 | ＞0.05 |
| Uric acid (umol/L) | 366.5±84.1 | 370.8±81.3 | 419.5±93.1 | 399.8±81.3 | ＞0.05 |

FMT= fecal microbiota transplantation; BMI= body mass index; PLT= platelets; Hb= hemoglobin; HOMA IR= homeostasis model assessment of insulin resistance. *P*, baseline FMT vs baseline non-FMT

Supplementary Table 2. Comparison of liver function and metabolic data in NAFLD patients with fatty liver

| characteristic | FMT group(n=47) | | Non-FMT group(n=28) | | | *P*^a^ *value* | *P*^b^ *value* |
| --- | --- | --- | --- | --- | --- | --- | --- |
|  | Before treatment | After  treatment | Before treatment | After  treatment | |  |  |
| ALT (U/L) | 26.1±15.2 | 25.5±14.4 | 34.2±43.3 | | 23.8±9.1 | 0.809 | 0.684 |
| AST (U/L) | 26.9±15.6 | 24.0±5.7 | 26.9±15.6 | | 24.0±5.7 | 0.714 | 0.399 |
| TBil (μmol/L) | 13.9±6.7 | 13.6±6.0 | 14.3±6.0 | | 15.0±8.0 | 0.883 | 0.473 |
| Albumin (g/L) | 41.5±4.0 | 40.7±4.5 | 40.7±4.3 | | 39.1±4.9 | 0.731 | 0.229 |
| TC (mmol/L) | 5.1±1.1 | 4.9±1.4 | 4.7±1.3 | | 4.9±1.6 | 0.260 | 0.401 |
| TG (mmol/L) | 2.1±1.8 | 2.1±1.5 | 2.2±1.7 | | 2.5±3.8 | 0.793 | 0.983 |
| LDL (mmol/L) | 2.9±0.9 | 2.9±1.1 | 2.5±0.9 | | 2.4±0.8 | 0.496 | 0.156 |
| HDL (mmol/L) | 1.4±2.0 | 1.3±0.3 | 1.1±0.3 | | 1.2±0.5 | 0.547 | 0.387 |
| FibroScan (db/m) | 278.3±45.4 | 263.9±34.3 | 265.5±46.1 | | 282.5±47.5 | ＜0.01 | 0.049 |

*P*^a^：FMT group *vs* non-FMT group；*P*^b^: Group of FMT: before FMT *vs* after FMT

ALT=alanine transaminase; AST=aspartate transaminase; TC=total cholesterol; TG=triglyceride; LDL=low density lipoprotein; HDL=high density lipoprotein.

Supplementary table 3. Relative percentage% and B / F ratio of each phylum（Phylum level）

| phylum | A1  (n=47) | A2  (n=47) | A3  (n=10) | *P value* | |
| --- | --- | --- | --- | --- | --- |
|  |  |  |  | A1 *vs* A3 | A2 *vs* A3 |
| *Bacteroidetes* | 35.1 | 40.9 | 58.0 | 0.021* | # |
| *Firmicutes* | 52.6 | 50.6 | 37.5 | # | # |
| *Proteobacteria* | 9.9 | 3.1 | 0.7 | 0.032* | # |
| *Fusobacteria* | 1.3 | 1.1 | 2.9 | # | # |
| *Actinobacteria* | 1 | 1.8 | 0.9 | # | # |
| B/F ratio | 0.7 | 0.93 | 1.54 | 0.032* | # |

The values in the table indicate the average percentage content (%). **P*＜0.05；***P*＜0.001；#*P*＞0.05. B/F= Bacteroiddetes/ Firmicutes ratio.

A1, prior to FMT; A2, post FMT; A3, healthy individuals

Supplementary Table 4. Relative percentage% of each family（Family level）

| classification | family | A1  (n=10) | A2  (n=10) | A3  (n=10) | *P value* | | |
| --- | --- | --- | --- | --- | --- | --- | --- |
|  |  |  |  |  | A1 *vs* A3 | A2 *vs* A3 | A1 *vs* A2 |
| *Bacteroidetes* | *Bacteroidaceae* | 9.8 | 15.9 | 29.4 | 0.043* | # | 0.018* |
|  | *Muribaculaceae* | 0 | 0.2 | 0.5 | 0.007* | # | 0.022* |
| *Firmicutes* | *Ruminococcaceae* | 18.7 | 22.1 | 45.7 | 0.012* | 0.033* | # |
|  | *Christensenellaceae* | 0 | 0.9 | 0.4 | 0.017* | # | 0.006* |

The values in the table indicate the average percentage content (%). **P*＜0.05；***P*＜0.001；#*P*＞0.05.

A1, prior to FMT; A2, post FMT; A3, healthy individuals

Supplementary table 5. Relative percentage% of each genus（Genus level）

| genus | A1  (n=10) | A2  (n=10) | A3  (n=10) | *P value* | | |
| --- | --- | --- | --- | --- | --- | --- |
|  |  |  |  | A1 *vs* A3 | A2 *vs* A3 | A1 *vs* A2 |
| *Bacteroides* | 8.2 | 15.5 | 27.3 | 0.043* | # | 0.018* |
| *Christensenellaceae R-7* | 0.1 | 0.7 | 0.2 | 0.017* | # | # |
| *Escherichia-Shigella* | 4.1 | 0.3 | 0.1 | 0.015* | # | 0.02* |
| *Family XIII UCG-001* | 0 | 0 | 0 | 0.041* | 0.016 | # |
| *metagenome* | 0 | 0.1 | 0.5 | 0.007* | # | # |
| *Prevotella 2* |  |  |  | 0.052 | # | 0.001* |
| *Paraprevotella* | 0 | 0.2 | 0 | 0.039* | # | 0.029* |
| *Ruminococcus 1* | 0.2 | 1.1 | 3.7 | 0.027* | 0.012* | # |
| *Tyzzerella 3* | 0 | 0 | 0.4 | 0.039* | # | # |
| *Tyzzerella 4* | 0.1 | 0 | 0 | 0.061 | # | # |
| *UBA1819* | 0.2 | 0 | 0 | # | # | 0.011* |
| *[Eubacterium] coprostanoligenes group* | 0.7 | 1.1 | 2.5 | 0.037* | # | # |
| *[Eubacterium] ruminantium group* | 0 | 0.4 | 0.8 | 0.03* | # | # |
| *Intestinimonas* | 0 | 0 | 0.19 | 0.025* | # | # |
| *Mitsuokella* | 0 | 0.5 | 0.2 | 0.019* | # | # |
| *Rikenellaceae RC9 gut group* | 0 | 0.2 | 0.3 | 0.025* | # | # |
| *Roseburia* | 0.8 | 1.4 | 3.3 | 0.04* | # | # |
| *Subdoligranulum* | 0.7 | 1.0 | 9.1 | 0.049* | 0.033* | # |
| *Erysipelatoclostridium* | 0.04 | 0 | 0.04 | # | 0.034* | # |

The values in the table indicate the average percentage content (%). **P*＜0.05；***P*＜0.001；#*P*＞0.05

A1, prior to FMT; A2, post FMT; A3, healthy individuals

Supplementary Table 6. Comparison of clinical data between lean NAFLD and obesity NAFLD.

| Characteristic | lean NAFLD (n=15) | | obesity NAFLD (n=32) | | | *P^a^ value* | *P^b^ value* |
| --- | --- | --- | --- | --- | --- | --- | --- |
|  | B1 | C1 | B2 | | C2 |  |  |
| Gender (male) | 8（53.3%） | | 17（53.1%） | | | ＞0.05 | / |
| Age (year) | 53.6±16.1 | | 57±12.6 | | | ＞0.05 | / |
| Hypertension | 4（26.7%） | | 19（59.4%） | | | 0.037 | / |
| diabetes | 3（20%） | | 12（37.5%） | | | ＞0.05 | / |
| dyslipidemia | 11（73.3%） | | 18（56.3%） | | | 0.213 | / |
| hyperuricemia | 4（26.7%） | | 11（34.4%） | | | ＞0.05 | / |
| BMI (kg/m^2^) | 22.8  （21.1-24.5） | 22.7（21.0-24.4） | | 29.5（25.7-33.3） | 27.0（22.8-31.2） | ＜0.001 | ＞0.05 |
| Fasting blood glucose (mmol/L) | 5.1±2.0 | 4.8±1.3 | 6.1±2.5 | | 5.7±1.5 | ＞0.05 | ＞0.05 |
| Fasting insulin (mU) | 9.2±4.0 | 10.3±3.1 | 16.8±9.9 | | 16.2±8.0 | 0.041 | ＞0.05 |
| HOMA IR | 2.5±1.5 | 2.3±1.6 | 4.4±3.2 | | 4.1±2.4 | 0.046 | ＞0.05 |
| Uric acid (umol/L) | 327.9±90.6  (female)  351.6±92.8  (male) | 338.0±111.4(female)  329.6±72.7  (male) | 366.3±90.5  (female) 378.3±93.1  (male) | | 397.3±70.5  (female) 391.5±69.6  (male) | ＞0.05 | ＞0.05 |
| ALT (U/L) | 29.7±37.1 | 21.2±5.2 | 25.1±15.7 | | 24.1±7.9 | ＞0.05 | ＞0.05 |
| AST (U/L) | 28.6±20.3 | 21.9±4.1 | 24.1±9.7 | | 23.7±7.7 | ＞0.05 | ＞0.05 |
| albumin (g/L) | 39.7±6.2 | 40.7±6.1 | 41.3±4.6 | | 40.6±3.9 | ＞0.05 | ＞0.05 |
| TBil (μmol/L) | 18.5±17.1 | 18.6±12.4 | 13.4±4.7 | | 14.7±5.6 | ＞0.05 | ＞0.05 |
| TC (mmol/L) | 5.1±1.4 | 5.1±1.2 | 5.3±2.4 | | 4.8±1.2 | ＞0.05 | ＞0.05 |
| TG (mmol/L) | 2.5±2.5 | 2.1±1.9 | 2.1±1.9 | | 1.9±1.0 | 0.038 | ＞0.05 |
| LDL (mmol/L) | 2.8±0.9 | 2.7±1.1 | 2.8±0.9 | | 2.8±1.1 | ＞0.05 | ＞0.05 |
| HDL (mmol/L) | 1.1±0.5 | 1.4±0.4 | 1.5±2.3 | | 1.2±0.3 | ＞0.05 | ＞0.05 |
| FibroScan (db/m) | 253.4±36.2 | 235.7±24.5 | 285.2±42.3 | | 279.5±44.5 | 0.025 | 0.029 |

*P^a^value*：B1 *vs* B2; *P^b^value:* group of lean NAFLD *vs* group of obesity NAFLD

B1, prior to FMT in lean NAFLD; C1, post to FMT in lean NAFLD; B2, prior to FMT in obesity NAFLD; C2, post to FMT in obesity NAFLD

Supplementary table 7. The gut microbiota with statistical difference between the lean NAFLD and the obesity NAFLD before FMT treatment, from "phylum" to "species" level, as follows: B1 vs B2

| classification | Gut microbiota | B1 | B2 | *P：B1 vs B2* |
| --- | --- | --- | --- | --- |
| plylum | *Actinobacteria* | 2.5 | 0.5 | 0.048* |
| order | *Desulfovibrionales* | 0.5 | 0.1 | 0.046* |
| class | *Tannerellaceae* | 7.3 | 1 | 0.040* |
| genus | *Prevotella 2* | 0 | 0.3 | ＜0.001** |
|  | *Lachnospiraceae NK4A136 group* | 0 | 0.4 | ＜0.001** |
|  | *Lachnospiraceae ND3007 group* | 0 | 0.2 | ＜0.001** |
|  | *[Eubacterium] coprostanoligenes group* | 0 | 1 | ＜0.001** |
|  | *Mitsuokella* | 0 | 0.3 | ＜0.001** |
|  | *Fusicatenibacter* | 0 | 0.3 | 0.02* |
| species | *Bacteroides coprocola DSM 17136* | 0 | 2.6 | ＜0.001** |
|  | *Bifidobacterium longum subsp. longum* | 0.9 | 0 | ＜0.001** |
|  | *uncultured Roseburia sp.* | 0 | 0.2 | ＜0.001** |

The values in the table indicate the average percentage content (%). *P＜0.05；**P＜0.001；#P＞0.05.

B1, prior to FMT in lean NAFLD; B2, prior to FMT in obesity NAFLD

Supplementary table 8. The gut microbiota with statistical difference between the lean NAFLD and the obesity NAFLD before FMT treatment, from "phylum" to "species" level, as follows: B1 vs C1, B2 vs C2

| classification | Altered gut microbiota | B1 | B2 | C1 | C2 | *P*1  B1 *vs* C1 | *P*2  B2 *vs* C2 |
| --- | --- | --- | --- | --- | --- | --- | --- |
| phylun | *Actinobacteria* | 2.5 | 0.5 | 1 | 0.2 | 0.021* | # |
| order | *Desulfovibrionales* | 0.5 | 0.1 | 0.3 | 0.1 | 0.043* | # |
|  | *Selenomonadales* | 2.8 | 5.9 | 13.1 | 4.3 | 0.021* | # |
|  | *Lactobacillales* | 0.21 | 0.04 | 0.61 | 0.21 | # | 0.054# |
| class | *Rikenellaceae* | 1.7 | 2.2 | 1.2 | 0.3 | # | 0.045* |
|  | *Veillonellaceae* | 1.0 | 5.2 | 11.9 | 3.6 | 0.021* | # |
|  | *Bacteroidaceae* | 40.6 | 20.2 | 35.7 | 21.1 | 0.021* | # |
| genus | *Prevotella 2* | 0 | 0.3 | 2.6 | 6.0 | 0.014* | 0.022* |
|  | *[Eubacterium] coprostanoligenes group* | 0 | 1 | 0.9 | 1.1 | 0.047* | # |
|  | *Bacteroides* | 51.5 | 20.0 | 38.1 | 15.9 | 0.021* | # |
|  | *[Ruminococcus] gnavus group* | 2.9 | 0.2 | 0 | 0 | 0.047* | # |
|  | *Ruminococcus 2* | 0.2 | 0.3 | 0 | 0.6 | # | 0.034* |
|  | *[Eubacterium] ruminantium group* | 0 | 0 | 0.9 | 1.1 | 0.047* | # |
|  | *Alistipes* | 1.1 | 2.2 | 0.8 | 0.3 | # | 0.045* |
| species | *human gut metagenome* | 0 | 0.1 | 1.5 | 0.1 | 0.047* | # |
|  | unclassified | 86.8 | 89.1 | 91.4 | 75.6 | 0.043* | # |

The values in the table indicate the average percentage content (%). **P*＜0.05；***P*＜0.001；#*P*＞0.05.

B1, prior to FMT in lean NAFLD; C1, post to FMT in lean NAFLD; B2, prior to FMT in obesity NAFLD; C2, post to FMT in obesity NAFLD
